# The Use of Molecular Analyses in Voided Urine for the Assessment of Patients with Hematuria

**DOI:** 10.1371/journal.pone.0077657

**Published:** 2013-10-15

**Authors:** Willemien Beukers, Raju Kandimalla, Diandra van Houwelingen, Hrvoje Kovacic, Jie-Fen D. Chin, Hester F. Lingsma, Lars Dyrskjot, Ellen C. Zwarthoff

**Affiliations:** 1 Department of Pathology, Erasmus MC, Rotterdam, The Netherlands; 2 Department of Public Health, Erasmus MC, Rotterdam, The Netherlands; 3 Department of Molecular Medicine, Aarhus University Hospital, Aarhus, Denmark; Biomedical Research Foundation, Academy of Athens, Greece

## Abstract

**Introduction:**

Patients presenting with painless hematuria form a large part of the urological patient population. In many cases, especially in younger patients, the cause of hematuria is harmless. Nonetheless, hematuria could be a symptom of malignant disease and hence most patients will be subject to cystoscopy. In this study, we aimed to develop a prediction model based on methylation markers in combination with clinical variables, in order to stratify patients with high risk for bladder cancer.

**Material and Methods:**

Patients (n=169) presenting with painless hematuria were included. 54 patients were diagnosed with bladder cancer. In the remaining 115 patients, the cause of hematuria was non-malignant. Urine samples were collected prior to cystoscopy. Urine DNA was analyzed for methylation of *OSR1*, *SIM2*, *OTX1*, *MEIS1* and *ONECUT2*. Methylation percentages were calculated and were combined with clinical variables into a logistic regression model.

**Results:**

Logistic regression analysis based on the five methylation markers, age, gender and type of hematuria resulted in an area under the curve (AUC) of 0.88 and an optimism corrected AUC of 0.84 after internal validation by bootstrapping. Using a cut-off value of 0.307 allowed stratification of patients in a low-risk and high-risk group, resulting in a sensitivity of 82% (44/54) and a specificity of 82% (94/115). Most aggressive tumors were found in patients in the high-risk group. The addition of cytology to the prediction model, improved the AUC from 0.88 to 0.89, with a sensitivity and specificity of 85% (39/46) and 87% (80/92), retrospectively.

**Conclusions:**

This newly developed prediction model could be a helpful tool in risk stratification of patients presenting with painless hematuria. Accurate risk prediction might result in less extensive examination of low risk patients and thereby, reducing patient burden and costs. Further validation in a large prospective patient cohort is necessary to prove the true clinical value of this model.

## Introduction

Hematuria is one of the most common symptoms in urological practice, as up to 20% of all urological visits are for hematuria [1]. Hematuria in the adult population can have different causes, e.g. urinary tract infections, urolithiasis, benign prostate enlargement (BPH), and urologic malignancies. A urological cancer is found in approximately 5% of patients presenting with microscopic hematuria and in around 20% of patients with macroscopic hematuria [2,3]. However, in up to 60% of the patients no source of bleeding is found. Thus, most important in the evaluation of hematuria is the discrimination between a malignant and non-malignant cause. As a consequence, patients will be subject to an extensive examination, including cystoscopy, cytology and imaging of the upper urinary tract. The sensitivity of cystoscopy for the detection of bladder cancer (BC) is high, ranging from 68-83% [4,5]. Yet, it is an invasive procedure, causing pain and discomfort. Cytology has a high specificity, but a poor sensitivity especially for the detection of low-grade BC. Therefore cytology is only used in combination with cystoscopy. 

The use of molecular markers in the assessment of hematuria could be of importance in order to reduce costs and to avoid invasive diagnostic procedures. However, none of the current investigated markers have a high enough sensitivity and specificity to accurately distinguish between malignant and non-malignant causes. 

Recently, we developed a combination of methylation markers for urine-based follow-up of bladder cancer patients. The sensitivity of the combination of markers was 74% for the detection of bladder cancer recurrences [6]. The aim of the current study is to investigate whether these markers could also be used to predict the risk of urothelial cell carcinoma (UCC) in patients presenting with painless microscopic and macroscopic hematuria. 

## Material and Methods

### Patients and urine samples

In this study, urine samples were included from patients presenting with painless microscopic or macroscopic hematuria. All urine samples were retrieved from a sample bank. This sample bank contained urine samples that were prospectively collected prior to cystoscopy between January 2007 and July 2012 at the Urology outpatient departments of Erasmus MC, Rotterdam and Aarhus University Hospital, Denmark. There were 54 urine samples available from which the cause of hematuria was malignant. As control group we included 115 hematuria samples with a non-malignant cause.

 All patients were examined by cystoscopy, computer tomography of the abdomen, and renal ultrasound. In addition, urine cytology was performed. Bladder tumors were biopsied and confirmed by histology. Urine samples were collected prior cystoscopy and were processed within 12-hours after collection. Urine samples were centrifuged for 10 minutes at 2000*g. Cell pellets were re-suspended in 1ml PBS and centrifuged for minutes at 3000*g. Supernatant was discarded and cell pellets were stored at -80°C until DNA isolation. DNA was extracted using the QIAamp mini and Blood kit (Qiagen) according to manufacturer’s protocol. Samples from Erasmus MC were used according to the code of secondary use of human tissue (www.federa.org). All patients were checked in the Erasmus MC opt-out objection system. In case of objection, patients were excluded from analysis. For minors, parents or caretakers should have filled in the statement of objection. Informed written consent was obtained from patients at Aarhus University Hospital and the study was approved by The Central Denmark Region Committees on Biomedical Research Ethics (1994/2920). 

### Methylation analysis

Recently, we developed a multiplex methylation assay in order to detect bladder cancer in voided urine [6]. This assay consisted of probes covering CpG-sites in five different genes, namely *OSR1, OTX1, ONECUT2, MEIS1* and *SIM*. Methylation analysis was performed using the EZ DNA Methylation-Gold™ Kit (Zymo Research Corporation, Irvine, California, USA) according to the manufacturer’s protocol. Briefly, DNA was treated with sodium bisulfite, followed by bisulfite-specific PCR for the five regions of interest. For each PCR reaction a DNA input of 20ng and PCR primer concentration of 20 pM was required. After the PCR, a Single Nucleotide Primer Extension (SNuPE) analysis was performed, using primers that annealed to the PCR product adjacent to the cytosine of interest. SNuPE probes were extended with a labeled dideoxynucleotide and the products were analyzed on an automatic sequencer (ABI PRISM 3100 Genetic Analyzer, Applied Biosystems), with the label indicating the presence or absence of a methylated cytosine. See Kandimalla et al for details on the primers and probes used [6]. For each gene, the methylation percentage was calculated by dividing the height of the methylated peak by the sum of the height of the methylated and unmethylated peaks multiplied by hundred. 

### Statistical analysis

Statistical analyses were performed using the Statistical Package for Social Sciences 20 (SPSS, Chicago) and R statistical Software for Statistical Computing (Vienna).

Univariable and multivariable logistic regression models were used to calculate the association between UCC and the predictor variables. The predictive accuracy of the model was determined by the area under the curve (AUC). The Bootstrap procedure was used for internal validation of the predictive model. P-values <0.05 were considered statistically significant. 

## Results

A total of 169 patients with painless microscopic or macroscopic hematuria were included in this study, comprising 104 men and 65 women. The mean age was 59 years (range 17-92). Patients and tumor characteristics are depicted in Table 1. In 54 cases, a bladder tumor was the cause of hematuria. In the remaining 115 patients, the cause of hematuria was non-malignant. Patients with UCC were significantly older and presented more frequent with macroscopic hematuria compared to patients with a non-malignant cause(p<0.001 and p=0.002). Cytology was performed in 81% of the patients. The sensitivity and specificity for cytology were 39% and 97, retrospectively. 

**Table 1:** Clinical and histopathological characteristics of 169 patients presenting with painless hematuria.

			**Hematuria UCC+**		**Hematuria UCC-**
			**n=54**		**n=115**
**Age**		*Mean (range)*	67 (29-92)		55 (17-86)
			**n (%)**		**n (%)**
**Sex**		*Male*	37 (69)		67 (58)
		*Female*	17 (31)		48 (42)
**Hematuria**		*microscopic*	18 (33)		68 (59)
		*macroscopic*	36 (67)		47 (41)
**Cytology**		*no tumor cells*	28 (52)		89 (77)
		*tumor cells*	18 (33)		3 (3)
		*not performed*	8 (15)		23 (20)
**Stage**		*Ta*	30 (55)		
		*T1*	7 (13)		
		*>=T2*	13 (24)		
		*Tx*	3 (6)		
		*Tis*	1 (2)		
**Grade**	***WHO1973***	*G1*	6 (11)		
		*G2*	11 (20)		
		*G3*	19 (35)		
		*Gx*	4 (7,5)		
	***WHO2004***	*Low Grade*	10 (19)		
		*High Grade*	4 (7,5)		

**Figure 1:**
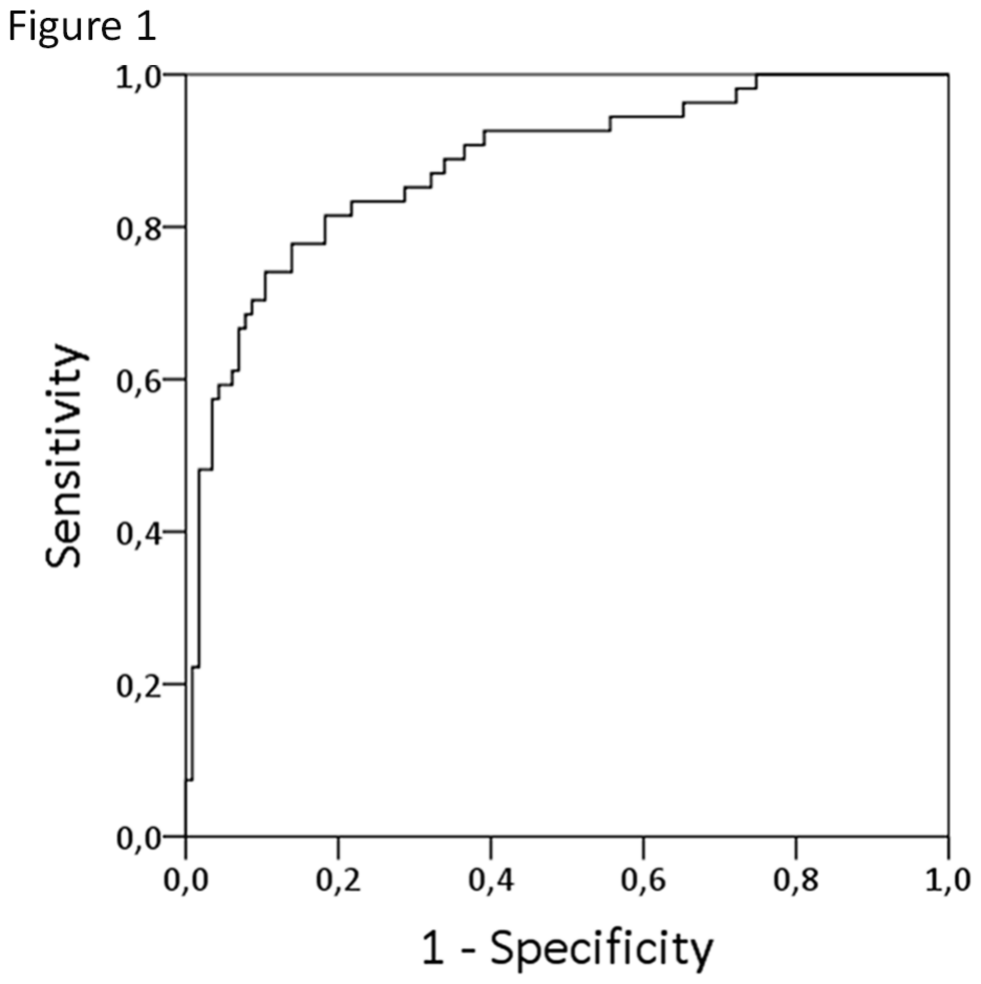
ROC of the multivariable model (AUC 0.88).

**Figure 2:**
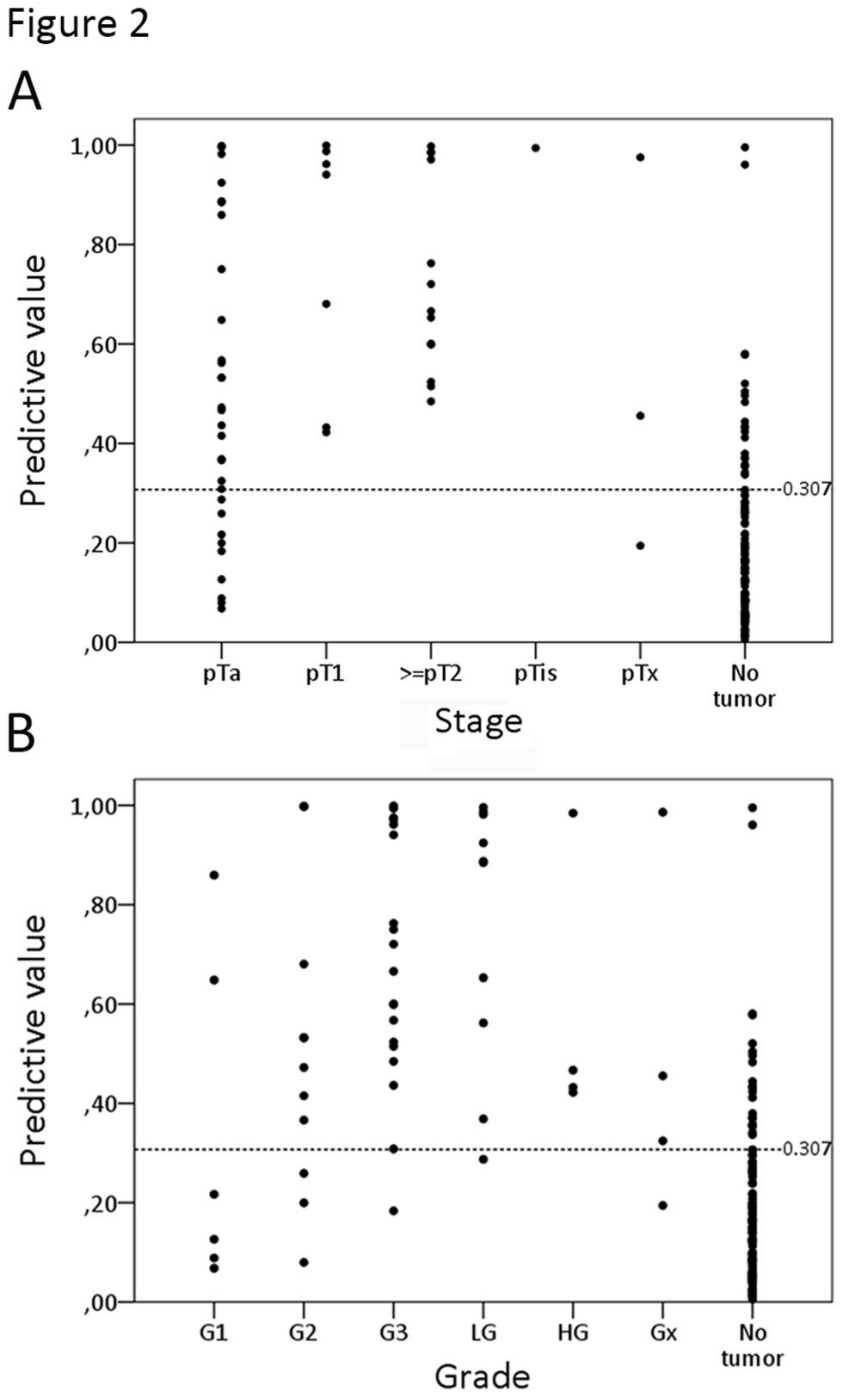
Scatterplot of risk values for the detection of urothelial cell carcinoma (UCC) in patients presenting with painless hematuria. X-axis depicts stage (A) and grade (B) of the resected tumors. Y-axis depicts the risk value based on the developed logistic regression model. Patients with risk value > 0.307 were considered at high-risk for having UCC. Patients with a risk value < 0.307 were considered at low-risk. Each dot represents a urine sample.

 Methylation analysis was performed for *OSR1*, *SIM2*, *OTX1*, *MEIS1* and *ONECUT2* and methylation percentages were calculated. Next, univariable logistic regression analysis was performed for the methylation percentages of the five different genes and the clinical variables age, gender, type of hematuria and cytology (Table 2). *OSR1*, *SIM2*, *OTX1*, *MEIS1, ONECUT2*, age, type of hematuria and cytology were all significant predictors for the presence of UCC. In order to calculate the combined effect, multivariable logistic regression analysis was performed. First a model was developed, based on the methylation percentages of the five genes and the clinical variables age, gender and type of hematuria. This resulted in an apparent AUC of 0.88 (Figure 1). In this model age, type of hematuria, *ONECUT2*, *OSR1* and *SIM2* were independent predictors for the presence of UCC (Table 3). After internal validation by bootstrapping, the optimism-corrected AUC was 0.84. To discriminate between patients at low-risk for UCC vs. patients at high-risk for UCC, a cut-off value with optimal sensitivity and specificity was determined. Based on a cut-off of 0.307, 44/54 of patients with UCC were in the high-risk group, resulting in a sensitivity of 82%. 94/115 (82%) of patients without a malignancy were in the low-risk group. In figure 2, the predictive values are shown according to stage (Figure 2A) and grade (Figure 2B) of the detected tumors. Tumors that were detected in patients in the low-risk group were mostly low stage and low grade. However, there was also one patient with a grade 3 tumor. On the other hand, all patients with a ≥pT2 tumor were in the high-risk group. In order to determine the additional value of cytology, a second model was developed. This model resulted in an AUC of 0.89 as shown in table 3 and an optimism-corrected AUC of 0.85. Based on a cut-off of 0.306, the sensitivity and specificity for the second model were 85% (39/46) and 87% (80/92), respectively.

**Table 2:** Univariable logistic regression analyses assessing the association between predictors and the presence of urothelial cell carcinoma.

	OR	95% CI	p value	AUC
Age (continuous)	1.062	(1.033, 1.092)	<0.001	0.72
Gender	0.641	(0.324, 1.270)	0.203	0.55
Type of hematuria	2.894	(1.033, 1.092)	0,002	0,63
Cytology	19.071	(5.229, 69.555)	<0.001	0.68
*OTX* ratio	1.061	(1.031, 1.093)	<0.001	0.69
*ONECUT* ratio	1.096	(1.050, 1.144)	<0.001	0.78
*OSR* ratio	1.072	(1.041, 1.103)	<0.001	0.75
*SIM* ratio	1.043	(1.021, 1.066)	<0.001	0.60
*MEIS* ratio	1.069	(1.034, 1.105)	<0.001	0.71

**Table 3:** Multivariable logistic regression analyses assessing the association between predictors and the presence of urothelial cell carcinoma.

**Multivariable model 1**				
	OR	95% CI	p value	AUC (%)
Age (continuous)	1.047	1.014, 1.081	0.005	88.1
Gender	0.924	0.376, 2.275	0.864	
Type of hematuria	3.743	1.521, 9.212	0.004	
*OTX* ratio	1.038	0.936, 1.152	0.478	
*ONECUT* ratio	1.117	1.015, 1.228	0.024	
*OSR* ratio	1.010	1.010, 1.124	0.020	
*SIM* ratio	0.829	0.829, 0.980	0.015	
*MEIS* ratio	0.986	0.919, 1.057	0.686	
**Multivariable model 2 (including cytology)**				
	OR	95% CI	p value	AUC (%)
Age (continuous)	1.032	0.997, 1.069	0.077	89.5
Gender	0.693	0.242, 1.989	0.496	
Type of hematuria	2.347	0.866, 6.361	0.094	
Cytology	10.956	2.269, 52.918	0.003	
*OTX* ratio	1.053	0.938, 1.181	0.384	
*ONECUT* ratio	1.085	0.981, 1.201	0.113	
*OSR* ratio	1.080	1.018, 1.145	0.010	
*SIM* ratio	0.875	0.786, 0.973	0.014	
*MEIS* ratio	0.988	0.907, 1.076	0.784	

## Discussion

Painless hematuria is a major problem in urological practice and the distinction between malignant and non-malignant causes is crucial. In this study, we developed a prediction model for the assessment of patients presenting with painless microscopic or macroscopic hematuria. With this newly developed model, the urologist will be able to adjust patient examination according to patient risk, resulting in a reduction of costs and patients discomfort. Previously, the five methylation markers were proven to be sensitive for the detection of recurrent UCC [6]. The methylation assay also appeared highly reproducible between different investigators. In the current study, the five markers discriminated with a high sensitivity between patients with and those without primary bladder cancer. Addition of the clinical variables age gender and type of hematuria increased the accuracy of the model. Age is one of the greatest risk factors for the development of bladder cancer and since men have a 3-4 times higher chance of developing UCC compared to women, gender also contributes significantly [7]. The use of cytology even more improved the diagnostic accuracy. Yet, we believe that the use of cytology as diagnostic test especially for the detection of low grade tumors is debatable. Therefore, we decided to calculate the fist prediction model without the addition of cytology. Inclusion of smoking history may even improve the predictive value further, since previous studies demonstrated that a history of smoking is an important independent predictor in the evaluation of hematuria [7-9]. However, smoking history was unavailable for the patients in this study. 

Up to now, multiple studies have been performed on the use of molecular tests in the diagnosis of bladder cancer in patients presenting with hematuria and some of these assays are already FDA approved [10-13]. However, due to suboptimal sensitivities and specificities, the analyses are mostly performed in addition to cystoscopy. Abogunrin et al. investigated whether biomarkers were able to improve the predictive power of a risk model which was based on clinical variables [14]. They considered the additional predictive value of nine different biomarkers to the prior predictive probability (PPP) that was based on age and smoking. They concluded that the addition of nuclear matrix protein 22 and vascular endothelial growth factor to the PPP improved the diagnostic accuracy from 0.76 to 0.90. In another study by Cha et al, the authors also combined molecular tests and clinical features into a multivariable regression model in order to predict the likelihood of having bladder cancer. They developed a nomogram based on the commercially available immunocytology assay (uCyt/ImmunoCyt), in combination with conventional cytology and clinical variables, i.e. age, gender, smoker, hematuria (microscopic vs. macroscopic) [15]. The AUC of this multivariable model was 0.904. However, cytology and immunocytology are highly dependent on the skills and experience of the pathologist [16]. 

A limitation of the current study was the retrospective design. Since this was not a consecutive series of patients, the composition of the patient cohort does not reflect true clinical practice. In addition, this model was internally validated by using the bootstrap method [17]. Therefore we suggest this model should be externally validated in a large prospective patient cohort of patients presenting with painless microscopic or macroscopic hematuria. 

In conclusion, we developed an accurate risk model for the evaluation of patients presenting with painless hematuria. Predicting the risk of bladder cancer in these patients could be of great value, resulting in less extensive examination of low risk patients and reduction of costs. Further validation in a large prospective patient cohort is necessary to prove the true clinical value of this newly developed model. 
